# *Miconia bullotricha* and *M. hirtistyla*, two new species of *Miconia* sect. *Lima* (Miconieae, Melastomataceae) from eastern Cuba

**DOI:** 10.3897/phytokeys.33.6766

**Published:** 2014-01-27

**Authors:** Lucas C. Majure, Eldis R. Bécquer, Walter S. Judd

**Affiliations:** 1 Department of Biology, University of Florida, Gainesville, Florida 32611–8525 U. S. A.; 2 Florida Museum of Natural History, University of Florida, Gainesville, Florida 32611–0575 U. S. A.; 3 Jardín Botánico Nacional, Universidad de La Habana, La Habana, Cuba

**Keywords:** Cuba, Greater Antilles, *Leandra*, *Ossaea*, Sierra Maestra

## Abstract

We describe two new species in *Miconia* sect. *Lima*, *Miconia bullotricha* Bécquer & Majure and *Miconia hirtistyla* Majure & Judd, from eastern, Cuba. We also provide illustrations and distribution maps for the two species, as well as a key to members of the *Lima* clade on Cuba.

## Abstract (es)

Describimos dos nuevas especies de *Miconia* sect. *Lima*, *Miconia bullotricha* Bécquer & Majure y *Miconia hirtistyla* Majure & Judd, del este de Cuba. También, proveemos ilustraciones y mapas de distribución para las dos especies, y una clave para distinguir los miembros del clado *Lima* en Cuba.

## Introduction

*Miconia* sect. *Lima* Majure & Judd (2013a), i.e., the *Lima* clade, is a small group of 18 species, including the two species described here. The clade occurs throughout the Greater Antilles, with the exception of Puerto Rico. Ten species are endemic to Cuba, seven are endemic to Hispaniola and one species occurs only on Jamaica. This clade is characterized by the production of bulla-based hairs on virtually all surfaces of the plant, including stems, petioles, leaf surfaces, inflorescence axes, bracts, hypanthia, and even the petal abaxial surfaces. The bulla-based hairs on the adaxial leaf surfaces often are very well developed and more or less cover the areoles. Long stemmed, clavate-dendritic hairs also are common on the adaxial leaf surface, especially toward the base of the blade, along the primary, secondary, and tertiary veins. Certain species within the clade also produce acarodomatia composed of tufts of multicellular hairs. Species within the clade have terminal inflorescences and typically flowers with ovate to elliptic petals with acute apices (Majure and Judd 2013a, b), which led the majority of species to be described (e.g., Alain 1955, Britton and Wilson 1920, Urban 1923, 1927, 1929, 1931) or recognized as either *Ossaea* or *Leandra* in previous treatments (Alain 1956, Liogier 2000, Judd and Skean 1991, Michelangeli and Bécquer 2012). The production of anthers with one dorso-basal appendage, and a single, dorsally-oriented pore, also is common in this clade (Majure and Judd 2013a), although not all species possess these features (Majure and Judd 2013b).

While working on a taxonomic revision of the *Lima* clade, two previously undescribed species from eastern Cuba were discovered, *Miconia bullotricha* Bécquer & Majure and *Miconia hirtistyla* Majure & Judd. We describe these two species herein and provide distribution maps, illustrations, and comparisons with other putative close relatives. We also provide a key to the species of *Miconia* sect. *Lima* on Cuba.

It is noteworthy that most members of the *Lima* clade are local endemics and this is especially true for numerous species from eastern Cuba, which are either found in the Sierra Maestra region (*Miconia hirtistyla*, *Miconia norlindii* (Urb.) Majure & Judd, *Miconia tentaculicapitata* Majure & Judd) or the mountains of the Baracoa and Moa regions (*Miconia bullotricha*, *Miconia cubacinerea* Majure & Judd,* M. granulata* (Urb.) Majure & Judd, *Miconia jashaferi* Majure & Judd). *Miconia argentimuricata* Majure & Judd is restricted to both the Sierra Maestra and Moa-Baracoa regions, and *Miconia ottoschmidtii* (Urb.) Majure & Judd is the only widespread species across the island. *Miconia cubana* (Alain) Majure & Judd is the only species endemic to western Cuba. Eastern Cuba is known for high endemism with 53.4 % of endemics to the island known only from the former Oriente Province (Borhidi 1991), which is now composed of the provinces of Granma, Guantánamo, Holguín, Las Tunas, and Santiago de Cuba. The cause for such high endemism in this region has been suggested to be the result of dramatic elevational diversity, as well as the diversity of soil types and other ecological and geographic factors (Borhidi 1991, López Almirall 2013). In fact, the highest number of endemics on Cuba is found in areas of the Nipe Mountains, Sierra de Cristal and Moa region, all of which are composed of serpentine soils. Approximately 31.2 % of all Cuban endemic plant species are found on serpentine soils (Borhidi 1991). Thus, it appears that specific soil types are certainly a direct driver of local endemism, along with topological relief, as well as other ecological factors, such as regional climate patterns. Such combined factors likely resulted in the high number of local endemics in the *Lima* clade of eastern Cuba. Numerous other groups show local endemism in these areas as well (e.g., *Lyonia* Nutt. sect.* Lyonia*, Judd 1981; *Heptanthus* Griseb., *Neobracea* Britt., *Oplonia* Raf., *Platygyne* Muell., Borhidi 1991; *Exostema* (Pers.) Bonpl., McDowell et al. 2003; *Miconia* sect. *Chaenopleura* (L.C. Rich. ex DC.) Hook., Judd 2007; the *Calycopteris* clade of *Miconia*, Judd & Majure 2013, Judd et al. in press).

## Key to the species of *Miconia* sect. *Lima* on Cuba

**Table:** 

1	Adaxial leaf surfaces appearing velvety or soft, covered in narrowly dilated (i.e., poorly developed bulla–based) hairs with long, attenuate apices, and hairs mostly of one size, usually not covering leaf areoles	2
–	Adaxial leaf surfaces appearing as a rasp or file, lizard or toad skin, covered in broadly dilated (i.e., well developed bulla-based hairs), with acute, attenuate to truncate apices, and hairs of several sizes, mostly covering the leaf areoles	5
2	Inflorescence an expanded, open, compound cyme, very delicate (inflorescence axis and branches 0.2–0.6 mm wide), with proximal inflorescence branches 8–25 mm long, bracteoles linear, 0.5–0.6 × 0.15–0.3 mm, calyx lobes and teeth 4, ovary 4-locular and 4 lobed, Pinar del Río	*Miconia cubana*
–	Inflorescence a dense cluster of sessile flowers (i.e., glomerulate), robust (inflorescence axis and branches 0.8–1.5 mm wide), proximal inflorescence branches 0–5.5 mm long, bracteoles foliose, ovate, obovate or orbicular, 2.8–4.3 × 1–2.4 mm, calyx lobes and teeth 4–7, ovary 3-locular and unlobed, Granma, Guantánamo, Holguín, Santiago de Cuba	3
3	Leaf apices narrowly acute to acuminate, leaf margins composed of large and small bulla-based hairs (appearing jagged), inflorescences pendant, anther pores dorsally oriented, Baracoa, Moa, Sierra de Cristal	*Miconia jashaferi*
–	Leaf apices broadly acute, leaf margins with one size of bulla-based hairs (appearing smooth), inflorescences erect, anther pores apically oriented, Baracoa and western Sierra Maestra	4
4	Abaxial leaf surface deeply pitted (to 0.5 mm deep) from the bulla-based hairs produced on the adaxial surface, styles pubescent, calyx teeth 4.5–4.6 × 0.2–0.4 mm, Sierra Maestra	*Miconia hirtistyla*
–	Abaxial leaf surface shallowly pitted (pits to <0.1 mm deep), styles glabrous, calyx teeth 5.7–6.2 × 0.6–0.7 mm, Baracoa	*Miconia cubacinerea*
5	Bulla-based hairs on adaxial leaf surface truncate, the larger hairs broadly spaced from one another, not meeting at the bases, and surrounded by smaller hairs, the smaller hairs forming a ring around the larger hairs, tertiary veins inconspicuous on the adaxial surface, stem hairs granulate	6
–	Bulla-based hairs on adaxial leaf surface with attenuate, acute or truncate apices, the larger hairs not appearing broadly spaced from one another and oftentimes nearly meeting at the bases, the smaller hairs not forming a ring around the larger hairs, tertiary veins conspicuous on adaxial surface, stem hairs long and shaggy or granulate	7
6	Leaves 3-veined, domatia absent, basal inflorescence branches sometime pendant, ovaries strongly 4-lobed, leaves narrowly ovate to narrowly elliptic, leaf length/width quotient (1.38–6.25), apices acute to acuminate, Sierra de Moa and Baracoa regions	*Miconia granulata*
–	Leaves 5-veined, domatia present at least at the junction of primary and secondary veins, basal inflorescence branches consistently erect, ovaries not strongly 4-lobed, leaves elliptic or narrowly elliptic, leaf length/width quotient (1.38–4.85), apices acute to obtuse, Sierra Maestra	*Miconia norlindii*
7	Adaxial leaf surface drying dark brown, bronze or silver colored, stem and hypanthia clothed in long, shaggy ascending hairs 0.9–4 mm long, mountains of eastern Cuba	8
–	Adaxial leaf surface drying light brown, green or yellow, stem and hypanthia clothed in short, spreading, slightly ascending or descending hairs 0.1–0.5 mm long, Guantánamo or otherwise widespread in Cuba	9
8	Leaves mostly elliptic, apices broadly acute to obtuse, abaxial leaf surface covered in short, appressed to slightly erect bulla-based hairs, the epidermis mostly obscured, inflorescence a 3–5 flowered condensed cyme, proximal inflorescence branches absent, bracts and bracteoles broad and foliaceous, Sierra Maestra	*Miconia tentaculicapitata*
–	Leaves mostly ovate, apices narrowly acute to acuminate, abaxial leaf surface covered in long, erect to spreading narrowly bulla-based hairs, the epidermis clearly seen, inflorescence a 3–42 flowered, open, compound cyme, proximal inflorescence branches 8–26 mm long, bracts and bracteoles oblong or ovate with attenuate apices, not foliaceous, Sierra Maestra and Sierra de Moa region	*Miconia argentimuricata*
9	Entire inflorescence usually pendant, floral buds globose, calyx teeth 1.75–2.2 mm long, abaxial leaf surface hairs erect throughout the lamina and along veins, stem indumentum generally with apices attenuate and strongly recurved upwards, innermost pair of secondary veins produced 2–6 mm from the leaf base, mountains of Guantánamo province	*Miconia bullotricha*
–	Inflorescence erect or occasionally with basal most branches pendant, floral buds quadrangular, calyx teeth 0.4–0.8 mm long, abaxial leaf surface hairs erect, spreading or appressed throughout the lamina, appressed to spreading along the veins, stem indumentum generally granulate with apices truncate or only short attenuate and recurved upwards or not, innermost pair of secondary veins produced 0.8–25 mm from the leaf base, widespread on Cuba	*Miconia ottoschmidtii*

## Systematics

### Miconia bullotricha

Bécquer & Majure sp. nov.

urn:lsid:ipni.org:names:77135720-1

http://species-id.net/wiki/Miconia_bullotricha

Figs 1 , 2

#### Diagnosis.

Species differing from *Miconia ottoschmidtii* in its stem indumentum generally with apices attenuate and strongly recurved upwards, (vs. more frequently granulate stem indumentum with apices truncate) more frequently ovate leaf shape (vs. mostly elliptic leaves), innermost pair of secondary veins produced 2–6 mm from leaf base (vs. 0.8–25 mm from leaf base), erect bulla-based hairs on lamina and tertiary veins of leaf abaxial surface (vs. mostly spreading to appressed bulla-based hairs), entire inflorescences pendant (vs. mostly erect inflorescences except for sometimes pendant basal inflorescence branches), globose floral buds (vs. quadrangular floral buds), and calyx teeth length (1.75–2.2 vs. 0.4–0.8 mm).

#### Type.

**Figure 1.**
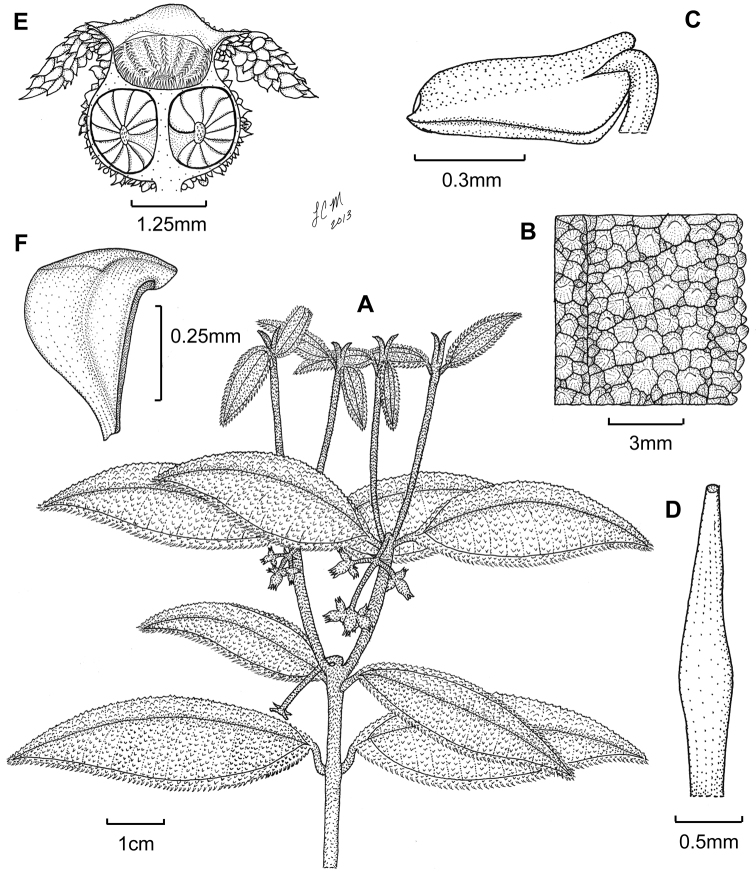
Illustration of *Miconia bullotricha*. **A** habit **B** close-up of leaf adaxial surface **C** immature stamen **D** style **E** fruit longitudinal section **F** seed (all from *Bisse et al. HFC-49930*).

CUBA. Guantánamo: Palenque. Bernardo. Sierra del Frijol, Loma Bernardo, 800–900 m, 21 May 1983, *Bisse J., Beurton C., Dietrich H., Gutiérrez J., Lepper L., Dolmus R., Köhler E., Rankin R., Arias I. HFC-49930* (holotype: HAJB!; isotypes: B 100362845!, HAJB!, JE!, NY!; Fig. 1).

#### Description.

Evergreen shrub (height unknown); stems round in cross section, not ridged, the internodes 1.1–2.4 cm long; stems densely covered in bulla-based hairs with strongly to narrowly dilated bases, to 0.3 mm long, the hairs spreading to descending with apices recurved upwards, young stem hairs often dark purple in color; nodal line present, inconspicuous. Leaves opposite, decussate, elliptic to ovate-elliptic, often slightly falcate, 4.2–6 × 1–2.2 cm, often slightly anisophyllous, yellowish when dried; apex narrowly acute; base rounded to broadly cuneate or abruptly cuneate; margin revolute, dentate, the dentations obscure, each covered in one large, bulla-based hair, venation acrodromous, 3 (–5) veined, 1 primary vein and 1 (rarely 2) pairs of suprabasal secondary veins, often asymmetrical at union with midvein, produced 2–6 mm from the leaf base, positioned 0.7–3 mm in from margin at widest part of blade, the tertiary veins percurrent, ± perpendicular to midvein, 2–3 mm apart at mid-leaf, intertertiary veins present, often joined by quaternary veins; adaxial leaf surface with primary and secondary veins impressed, tertiary veins flat to slightly impressed, remaining veins flat, abaxial surface with primary, secondary and tertiary veins raised, the higher order veins ± flat to slightly raised (i.e., clearly visible to more or less obscure); adaxial leaf surface completely covered in erect bulla-based hairs, these fully expanded at the base, thus the lamina obscured, widest hair bases to 1.5 mm wide, hair apices acute to truncate, sometimes slightly recurved toward the leaf margin, sessile, glandular hairs occurring between the bases of bulla-based hairs; abaxial leaf surface nearly completely covered with bulla-based hairs with strongly to narrowly dilated bases, the lamina areoles not completely filled, the hairs along the epidermis erect with apices recurved or not, veins completely covered by spreading to erect hairs mostly with narrowly dilated bases and recurved apices, sessile, glandular hairs occurring throughout the lamina, as well as along veins; domatia inconspicuous, of multicellular, tufts of linear hairs present in the axils of the primary and secondary, as well as primary and tertiary veins; petiole 5–8 mm long, covered in spreading bulla-based hairs, those of the adaxial surface slightly longer and narrower than those of the abaxial surface and recurved towards to the leaf blade. Inflorescences terminal, well-developed to reduced cymes of 3–13 flowers, 2–3.5 × 1.8–3.4 cm, the flowers produced in 3–7 flowered dichasia, the peduncle 0.7–1.4 cm long, usually conspicuously reflexed at base, thus the entire inflorescence pendant, the proximal inflorescence branches 0.5–1 cm long; bracts oblong to narrowly ovate, 1.1–2 mm long; bracteoles narrowly ovate, ca. 0.5–0.7 × 0.2-0.3 mm, glabrous or with small bulla-based hairs at base, bracteoles generally resembling one large, bulla-based hair. Flowers perfect, actinomorphic, 4-merous, with pedicels 0-1 mm long. Hypanthium ca. 1.6 × 2.8 mm, ± globose, slightly constricted below torus, abaxial surface covered in granulate, bulla-based hairs with dilated bases and attenuate to truncate apices, to 0.5 mm long, and sessile, glandular hairs, the free portion of hypanthium 0.5–0.7 mm long, adaxial surface longitudinally ridged and covered by bulla-based hairs; calyx teeth 1.75–2.2 × 0.5 mm, linear and terete, recurved upon maturation, covered in bulla-based hairs; calyx lobes ± triangular, apex acute, ca. 1 × 1.3 mm, with bulla-based hairs abaxially and sessile, glandular hairs produced adaxially; calyx tube not tearing, ca. 0.4 mm long, with bulla-based hairs abaxially, sessile, glandular hairs adaxially and clavate-dendritic hairs produced at the apex; petals 4, (i.e., only seen in bud), ovate to elliptic with acute apices, apices with one, slightly bulla-based hair produced subapically, hair to 0.5 mm long; stamens 8 (immature), filaments glabrous, anthers ovate, with a well-developed dorso-basal appendage and one apically-oriented pore (the pore position could be an artifact of level of maturity); style (immature) dilated in the middle, subtended by a short crown of multicellular hairs, these only slightly longer than the surrounding bulla-based hairs on the ovary apex; stigma punctate; ovary ca. 1.4 × 2.4 mm, apex flat, with bulla-based hairs, 4 locular, with axillary placentation, the placenta deeply intruded into locule; berries (immature) globose, ca. 3–3.4 × 3 mm; seeds (immature) 0.2–0.6 mm long, obpyramidal, testa smooth, light brown, raphe extending the length of the seed, dark brown.

#### Distribution and habitat.

**Figure 2.**
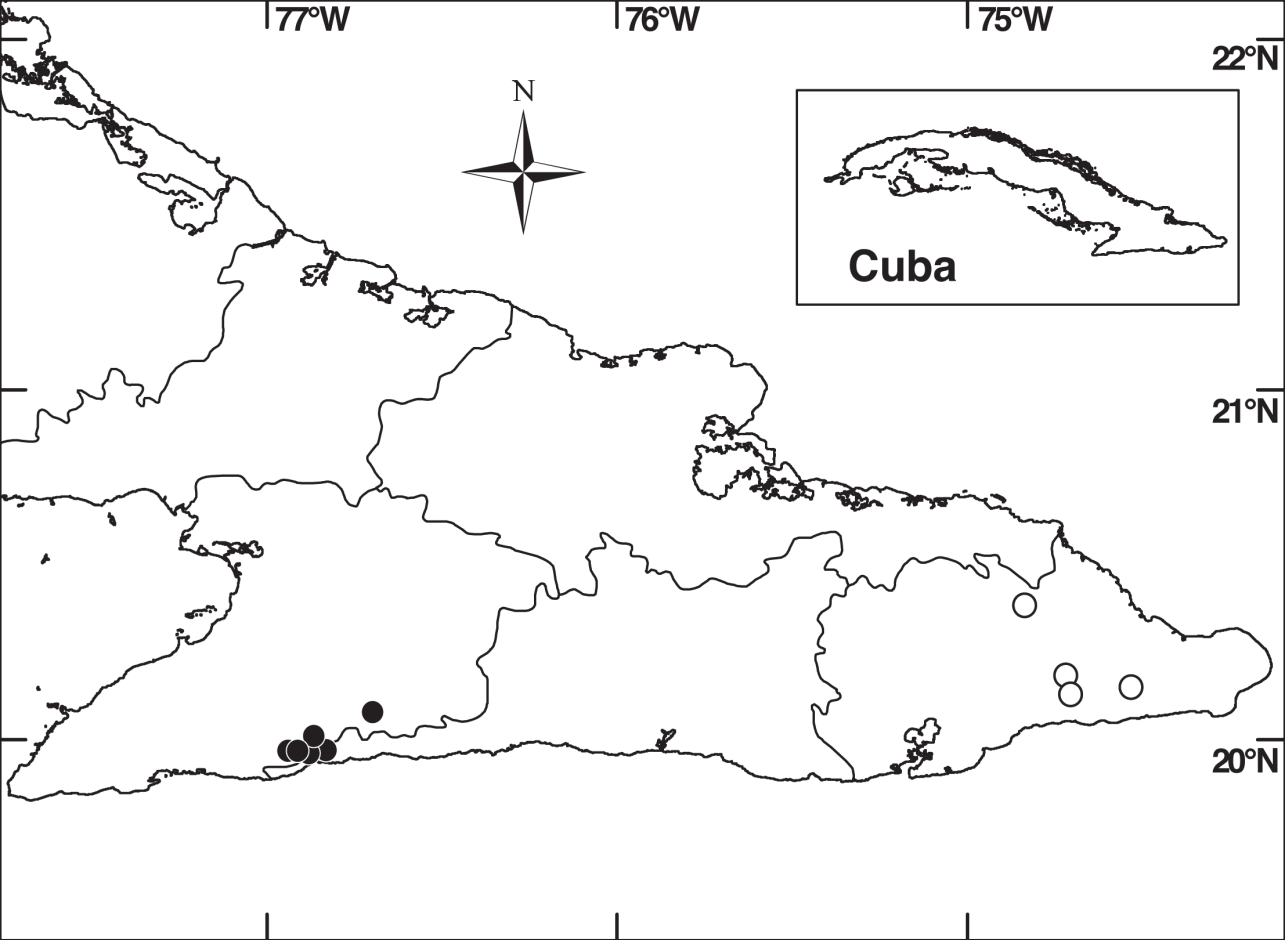
Distribution of *Miconia hirtistyla* in Granma and Santiago de Cuba provinces (closed circles) and *Miconia bullotricha* in Guantánamo Province (open circles).

*Miconia bullotricha* is endemic to eastern Cuba (province of Guantánamo; Fig. 2), where it occurs in semi-dry, montane and elfin forest on serpentine soils at elevations of 500–1000 m. Associated melastomes include *Calycogonium grisebachii* Triana, *Miconia baracoensis* Urb. and *Ossaea pauciflora* (Naudin) Urb.

#### Phenology.

Plants with buds and young fruits have been collected in May.

#### Etymology.

The specific epithet “*bullotricha*” refers to the well-developed bulla-based hairs on the adaxial leaf surface. Although *bulla* is Latin in origin, we base formation of our compound epithet on the Greek rules for connecting vowels. Thus, we use “o” here instead of “i”, as we find “*bullotricha*” to be more euphonious than “*bullitricha*.” The connecting vowel “o” has had widespread usage in classical Latin based on the large influence of Greek (Stearn 1966).

#### Conservation status.

We do not have extensive knowledge of population level numbers of individuals or the reproductive biology of this species, so the conservation status of *Miconia bullotricha* cannot be critically evaluated at this time. More fieldwork is imperative to assess the status of this species. However, deforestation has occurred in the surrounding areas from where *Miconia bullotricha* is known, and thus, the species most likely should be considered threatened by habitat loss and other anthropogenic disturbances.

#### Specimens examined.

**Cuba.** **Guantánamo:** Baracoa. Imías, Sierra de Imías, loma Jubal (al sur de Los Lechugos), 900–1000 m, 19 Aug 1975, *A. Álvarez de Zayas & al. HFC-27626* (B, HAC, HAJB, JE); Baracoa. Sierra de Purial, La Gurbia, 700 m, May 1968, *J. Bisse & L. Rojas HFC-8562* (HAJB); IBID, *HFC-9389* (HAJB); Baracoa. falda suroeste de la loma del Mirador, 500 m, 9 Aug 1975, *J. Bisse & F. K. Meyer HFC-27230* (B, HAC, HAJB, JE); Yateras Palenque. Sierra del Frijol, cerca de Bernardo, 800 m, 17 May 1983, *J. Bisse & al. HFC-49721* (B, JE); IBID, *HFC-49731* (HAJB).

#### Discussion.

*Miconia bullotricha* likely belongs to a subclade within the *Lima* clade that contains the phenetically similar, Cuban endemic, *Miconia ottoschmidtii*, as well as other members of the *Miconia lima* complex (Majure et al. 2013b, Majure et al. unpubl. data). These species are recognized by their very well developed bulla-based hairs on the upper leaf surface (which mostly cover the leaf areoles; Fig. 1), as well as expanded, pyramidal inflorescences consisting of cymose clusters of flowers subtended by highly reduced bracts. The only exception to this is *Miconia pedunculata* Majure & Judd of the Cordillera Central, Dominican Republic that has widely spaced bulla-based hairs on the adaxial leaf surface, which do not completely fill the leaf areoles, and flowers that are subtended by foliaceous bracts.

As mentioned above, *Miconia bullotricha* is phenetically most similar to *Miconia ottoschmidtii*. However, the two species can be easily distinguished by stem and leaf indumentum, where *Miconia bullotricha* has the stem indumentum generally with apices attenuate and strongly recurved upwards, (as opposed to granulate with the apices truncate or short attenuate in *Miconia ottoschmidtii*; although it should be noted that central and northern Cuban populations of *Miconia ottoschmidtii* have a tendency towards stem hairs with longer, attenuate apices that may be recurved upwards), and erect bulla-based hairs throughout the lamina and along the tertiary veins on the leaf abaxial surface (while *Miconia ottoschmidtii* has spreading to appressed bulla-based hairs throughout the lamina and along the tertiary veins of the leaf abaxial surface). *Miconia bullotricha* usually produces entirely pendant inflorescences (Fig. 1), in contrast to the erect inflorescences of *Miconia ottoschmidtii* and the rest of the members of the subclade (however, several species often produce pendant basal inflorescence branches, including *Miconia ottoschmidtii*), and has longer calyx teeth than *Miconia ottoschmidtii* (1.75–2.2 vs. 0.4–0.8 mm). Also, *Miconia bullotricha* has globose floral buds, while *Miconia ottoschmidtii* exhibits quadrangular floral buds.

*Miconia bullotricha* adheres to the morphological/phenetic and diagnostic species concepts (Judd 2007, Wheeler and Platnick 2000), and considering the putative autapomorphy of entirely pendant inflorescences, is likely a cladospecies (Donoghue 1985).

### Miconia hirtistyla

Majure & Judd sp. nov.

urn:lsid:ipni.org:names:77135721-1

http://species-id.net/wiki/Miconia_hirtistyla

Figs 2 , 3

#### Diagnosis.

Species differing from *Miconia jashaferi* in having erect inflorescences, clawed petals, apically oriented anther pores and pubescent styles. Species differing from *Miconia cubacinerea* in having pubescent styles, clawed petals, and shorter calyx teeth (4.5–4.6 mm in *Miconia hirtistyla* vs. 5.7–6.2 mm in *Miconia cubacinerea*).

#### Type.

**Figure 3.**
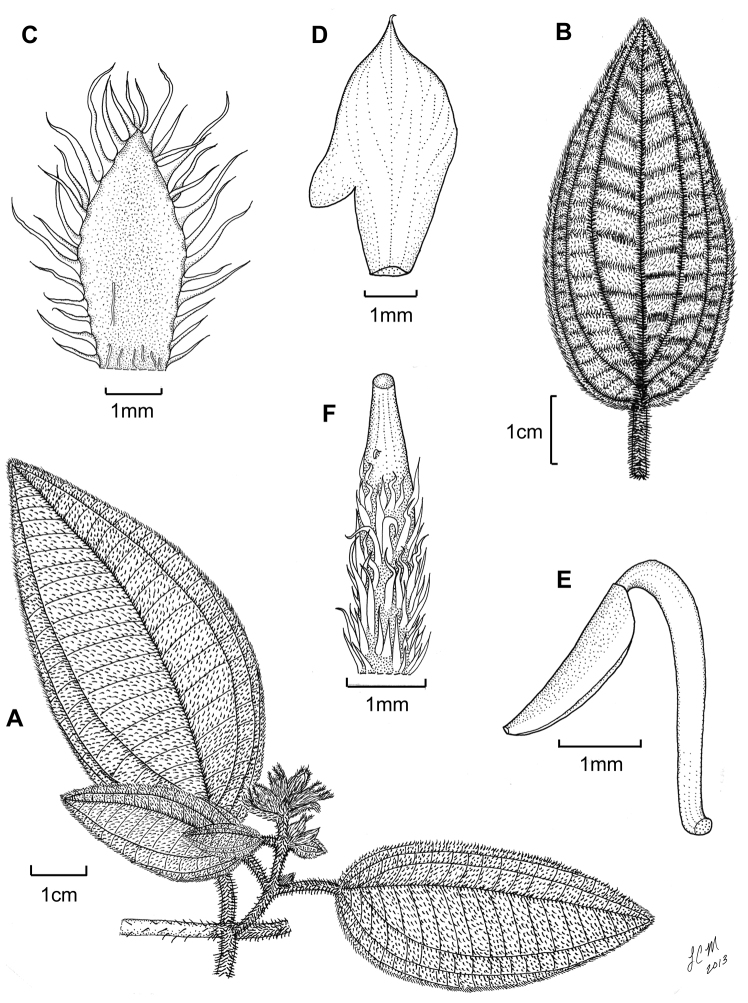
Illustration of *Miconia hirtistyla*. **A** habit (*Ekman 14617*) **B** leaf abaxial surface (*León LS-10923*) **C** bracteole (*León LS-10923*) **D** petal (*Ekman 14617*) **E** stamen (*Ekman 14617*) **F** style (*Ekman 14617*).

CUBA. Santiago de Cuba: Southern Oriente and Pico Turquino, high [Sierra] Maestra, July 1922, *Fre. León LS-10923* (holotype: NY!; isotype: GH!, HAC!; Fig. 3).

#### Description.

Evergreen shrub (height unknown); stems round in cross section, not ridged, the internodes 0.4–3.3 cm long, stem indumentum of bulla-based hairs to 1.6 mm long, these shaggy, spreading to slightly descending; nodal line absent. Leaves opposite, decussate, ovate to elliptic, not falcate, 1.6–8.2 × 1.4–3.9 cm, slightly to strongly anisophyllous (larger leaves at a node to twice as large as the smaller leaf), dark brown when dried, apex broadly acute, base broadly acute to rounded, margin dentate, dentations obscure, each covered in one large bulla-based hair, venation acrodromous, 7 veined, the midvein and 3 pairs of arching secondary veins, secondary veins mostly basal, the innermost pair, suprabasal, produced 3–9 mm from leaf base, positioned 2.5–11 mm in from margin at widest point of blade, tertiary veins percurrent, ± perpendicular to midvein, 1.5–4.1 mm apart at midleaf, intertertiary veins present, tertiary veins often joined by quaternary veins; adaxial leaf surface with primary, secondary and tertiary veins impressed, quaternary veins obscure, abaxial surface with all veins conspicuously raised; adaxial leaf surface covered in well developed but narrow bulla-based hairs mostly but not entirely covering the leaf areoles, widest hair bases to 0.8 mm, apices of bulla-based hairs mostly erect to recurved, sessile, glandular, hairs produced along the primary, secondary, tertiary, and quaternary veins between the bulla-based hairs; abaxial leaf surface covered in bulla-based hairs, these mostly erect with undulate apices, those along the primary, secondary, and tertiary veins spreading and larger than hairs produced throughout the lamina, lamina appearing as a series of pits from depressions of the bulla-based hairs produced from the upper leaf surface (i.e., foveolate), sessile, black, glandular hairs produced along all major and minor veins, domatia of tufts of multicellular, linear hairs abundant in axils of primary and secondary veins, as well as the axils of the primary and secondary with tertiary veins; petioles 0.4–1.8 cm long, covered in spreading, bulla-based hairs on both surfaces. Inflorescences terminal, cymose, 2–5 flowered, 1.3–2.4 × 1.2–3.8 cm, the flowers mostly produced in glomerulate clusters, the peduncle 0.6–1.3 cm long, proximal inflorescence branches 0.8–1.1 mm long, pedicels absent; bracts ovate to elliptic, foliaceous, 5–17 mm long; bracteoles foliaceous, elliptic, 2.8–4.3 × 1.7–2.1 mm, covered in bulla-based hairs marginally and abaxially and glabrous abaxially or with filiform hairs towards the base. Flowers perfect, actinomorphic, 6-merous, sessile. Hypanthium 2.6–3.2 mm long, short-oblong to globose, unlobed, slightly constricted below the torus, free portion of the hypanthium 1–1.4 mm long, abaxial surface covered in bulla-based hairs to 2.3 mm long, and occasional, sessile, glandular hairs near the bases of the bulla-based hairs; adaxial surface (i.e., free portion) covered in small, bulla-based hairs; calyx teeth 6, 4.5–4.6 × 0.2–0.4 mm, ascending or spreading, covered in bulla-based hairs; calyx lobes 6, ± triangular, apices acute, 1–1.4 × 1–1.5 mm, covered in bulla-based hairs abaxially and gland-headed, filiform hairs adaxially; calyx tube not tearing, 0.3–0.5 mm long with bulla-based hairs abaxially and sessile, glandular hairs, as well as filiform, gland-headed hairs adaxially and along the apex of the tube; petals 6, most likely white, elliptic to obovate, 5.7–6.6 × 2.7–3.1 mm, with an acuminate apex, only slightly to conspicuously clawed, with one slightly bulla-based hair produced abaxially, subapically, or in some cases, marginally, to 0.1 mm long; stamens 12; filaments 3.8–4.1 mm long, glabrous, anthers 2.2–2.6 mm long, ovate, with one apically oriented pore, anther thecae 2–2.5 mm long, anthers without a dorso-basal appendage; style 3.8–4.4 mm long, pubescent (i.e., with scattered, slightly bulla-based hairs), oblong to only slightly dilated in the middle, collar absent, style subtended by multicellular, linear to elongate-triangular (needle-like) hairs, which grade into the surrounding bulla-based hairs of the ovary apex, stigma punctate; ovary 1.2–2.8 × 1.5–2.5 mm, apex convex, with bulla-based hairs, placentation axile, placenta apparently not deeply intruded, 3-locular; berries not seen, mature seeds not seen.

#### Distribution and habitat.

*Miconia hirtistyla* is only known from the western Sierra Maestra, Cuba (Fig. 2), where it occurs in montane rainforest, pine forest and elfin forest on rocky soils at elevations of 700–1800 m. Associated melastomes include *Miconia argentimuricata*, *Miconia norlindii* and *Miconia nystroemii* Urb.

#### Phenology.

*Miconia hirtistyla* was collected in bud, at anthesis, and in immature fruit in March and July.

#### Etymology.

The specific epithet “*hirtistyla*” refers to the pubescent style of this species (Fig. 3). Within the *Lima* clade, *Miconia hirtistyla* is the only species that demonstrates this character.

#### Conservation status.

*Miconia hirtistyla* is mostly known from the very well protected forests of Turquino National Park. Although the species has not been collected since 1978, and we know nothing regarding its reproductive biology or population numbers, it is most likely not threatened by anthropogenic disturbance and habitat loss, at least in the areas immediately surrounding the park. Fieldwork will be necessary to appropriately assess the conservation status of this species.

#### Specimens examined.

**Cuba.** **Granma:** A lo largo del camino de Minas del Frio a Montpie, 23 Apr 1978, *J. Bisse et al. HFC-37347* (HAJB); Valle del arroyo Escondido, 700–1000 msm, 26 Apr 1978, *J. Bisse et al. HFC-37628* (HAJB); Bartolomé Masó. Estribo del Pico Turquino, 20 Apr 1979, *J. Bisse et al. HFC-40517* (HAJB); Manguito, pinares de la loma La Botella, 1200–1400 msm, 22 Mar 1970, *H. Lippold HFC-16283* (HAJB). **Santiago de Cuba:** Oriente, Pico Turquino, 12–26 Jul 1936, *J. Acuña SV-10189* (HAC); Oriente, Sierra Maestra, Cima del Pico Turquino, 10 July 1936, *J. Acuña SV-22705* (HAC); Oriente, Sierra Maestra, steep rocks of Loma Regino, 25 Jul 1922, *E.L. Ekman 14617* (S); southern Oriente and Pico Turquino, high [Sierra] Maestra, Jul 1922, *Fre. León LS-10927* (GH, NY).

#### Discussion.

**Figure 4.**
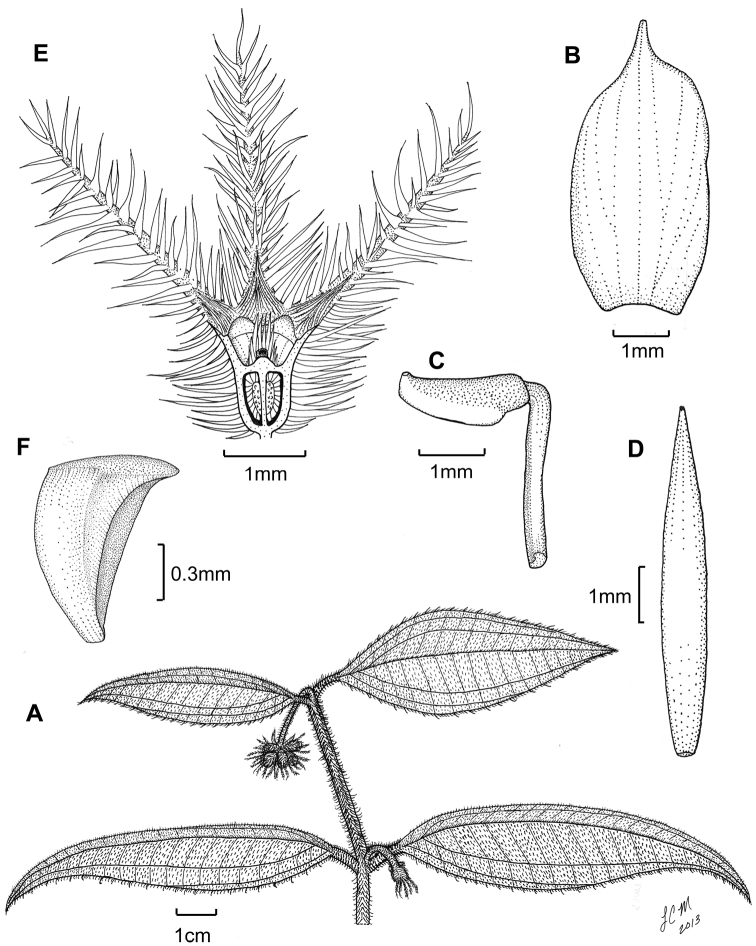
Illustration of *Miconia jashaferi*. **A** habit (*Ekman 3849*) **B** petal (*Alain 871*) **C** stamen (*Alain 871*) **D** style (*Alain 871*) **E** immature fruit longitudinal section (*Alain 871*), seed (*Acuña SV-13275*).

*Miconia hirtistyla* is the only species in the *Lima* clade known to possess pubescent styles and it is one of two species that exhibits clawed petals (e.g., *Miconia phrynosomaderma* Majure & Judd; Majure and Judd 2013a, a putatively distantly related species). Both characters are likely autapomorphies of *Miconia hirtistyla*, because morphology suggests that *Miconia phrynosomaderma* is more closely related to *Miconia limoides* and relatives (e.g., well-developed bulla-based hairs on leaf adaxial surface, open, expanded, cymose inflorescences, presence of anther dorso-basal appendages; see Majure and Judd 2013). *Miconia hirtistyla* is most likely closely related to *Miconia jashaferi* (Fig. 4), with which it had been confused, as well as *Miconia cubacinerea* and *Miconia tentaculicapitata*. All of these species have condensed inflorescences, leaf-like bracts and bracteoles, broad, oblong to obovate petals, a crown of long, needle-like hairs on the ovary apex and surrounding the style or merely needle-like hairs produced throughout the ovary apex, long, filiform, eglandular or gland-headed hairs along the calyx lobe adaxial surface and apex of calyx tube, long calyx teeth, as well as “shallowly” intruded placenta (versus deeply intruded placenta as in most other species of the *Lima* clade). All four species also lack a dorso-basal anther appendage, the presence of which otherwise is a common feature in the clade (Majure and Judd 2013a). *Miconia hirtistyla* differs from all three of these species by the presence of pubescent styles and clawed petals and from *Miconia jashaferi* and *Miconia cubacinerea* by hypanthium shape (short oblong to globose in *Miconia hirtistyla* vs. narrowly oblong to cylindrical in the latter two species). The species also differs from *Miconia jashaferi* in inflorescence structure (erect vs. pendant inflorescences; Figs 3, 4), leaf shape (elliptic to ovate with broadly to narrowly acute apices rather than mostly ovate with acuminate apices), anther size (2.2–2.6 mm long in *Miconia hirtistyla* vs. 1.8–2 mm long in *Miconia jashaferi*; Figs 3, 4) and shape (ovate with anther thecae continuous with sterile portion of anther vs. elliptic with anther thecae discontinuous with sterile portion of anther; Figs 3, 4), as well as having apically oriented anther pores instead of dorsally oriented pores. *Miconia hirtistyla* differs from *Miconia cubacinerea* in the pubescence of the abaxial leaf surface, in that *Miconia cubacinerea* has a clearly visible epidermis as a result of a sparser indumentum, while the epidermis of *Miconia hirtistyla* is mostly concealed by dense bulla-based hairs. Likewise, the primary, secondary, tertiary, and quaternary veins of *Miconia hirtistyla* are densely clothed in spreading bulla-based hairs, however, in *Miconia cubacinerea* the veins are easily seen, as the bulla-based hairs are less dense. The abaxial leaf surface of *Miconia cubacinerea* also is densely covered in sessile, glandular hairs, while that of *Miconia hirtistyla* has sparse, glandular hairs. The lamina of the abaxial leaf surface of *Miconia hirtistyla* is conspicuously, deeply pitted (as a result of the bulla-based hairs on the upper leaf surface to 0.5 mm deep), while that of *Miconia cubacinerea* is not deeply pitted (i.e., the pits are only superficial to <0.1 mm deep). The two species also differ in calyx teeth length (4.5–4.6 mm in *Miconia hirtistyla* vs. 5.7–6.2 mm in *Miconia cubacinerea*), and by the lack of clavate-dendritic hairs on the leaf adaxial surface and calyx teeth in *Miconia hirtistyla*.

Lastly, *Miconia hirtistyla* differs from *Miconia tentaculicapitata* by the less well-developed bulla-based hairs on the leaf adaxial surface, spreading to descending stem hairs, and lack of clavate-dendritic hairs on the leaf adaxial surface, as opposed to the well developed bulla-based hairs covering the leaf adaxial surface areoles, the ascending-appressed stem hairs, and presence of clavate-dendritic hairs on the leaf adaxial surface of *Miconia tentaculicapitata*.

*Miconia hirtistyla*, and the less phenetically similar, *Miconia tentaculicapitata*, are found in the western Sierra Maestra, while those species that are more phenetically similar to *Miconia hirtistyla*, i.e., *Miconia cubacinera* and *Miconia jashaferi*, are found in northeastern Cuba in the Sierra de Baracoa and Sierra de Moa regions; *Miconia jashaferi* also is found in the southern part of Sierra de Cristal.

*Miconia hirtistyla* is most likely a cladospecies (Donoghue 1985), as indicated by the putative autapomorphies of pubescent styles and clawed petals. The species also adheres to the morphological/phenetic species (Judd 2007) and diagnostic species concepts (Wheeler and Platnick 2000).

## Supplementary Material

XML Treatment for Miconia bullotricha

XML Treatment for Miconia hirtistyla
